# Impact of Modifiable Cardiovascular Risk Factors and Protective Therapies on Hemorrhagic Events in Cerebral Cavernous Malformations: A Systematic Review and Meta‐Analysis

**DOI:** 10.1002/brb3.71829

**Published:** 2026-10-08

**Authors:** Sai Sanikommu, Alejandro N. Santos, Bashar Dawoud, Josiah J. Z. Sherman, Christoph Wipplinger, Emade Jaman, Jayro Toledo, Ahmed Abdelsalam, Victor M. Lu, Alick Wang, Robert M. Starke

**Affiliations:** ^1^ Department of Neurological Surgery University of Miami School of Medicine Miami Florida USA; ^2^ Department of Neurological Surgery Cleveland Clinic Weston Weston Florida USA

**Keywords:** antithrombotics, cavernoma, cerebral cavernous malformation, hemorrhage, meta‐analysis, modifiable risk factors, obesity, smoking, statins, systematic review

## Abstract

**Background:**

Cerebral cavernous malformations (CCM) are vascular lesions prone to hemorrhage, but the influence of modifiable cardiovascular risk factors and commonly prescribed therapies on bleeding risk remains unclear.

**Methods:**

We performed a systematic review and meta‐analysis according to PRISMA 2020. PubMed/MEDLINE, Embase, and Scopus were searched from inception through August 31, 2025. Eligible observational studies and randomized trials reported adults with CCM stratified by modifiable cardiovascular risk factors or systemic therapies. Hemorrhagic presentation at diagnosis and prospective symptomatic hemorrhage or rebleeding during follow‐up were extracted and interpreted as distinct clinical endpoints. Comparable adjusted and crude estimates were pooled separately using random‐effects models; randomized trials with non‐comparable populations or endpoints were summarized narratively. Risk of bias and certainty of evidence were assessed using design‐appropriate Cochrane tools and GRADE, respectively.

**Results:**

Of 1275 records identified, 30 full texts were reviewed, and 22 studies met inclusion criteria, comprising 7331 patients with CCM. Most pooled analyses evaluated hemorrhagic presentation at diagnosis. Smoking was associated with increased odds of hemorrhagic presentation (aOR 1.41, 95% CI 1.03–1.93), and obesity showed an uncertain signal toward higher odds (aOR 1.77, 95% CI 1.00–3.13). Antithrombotic therapy was associated with lower odds of hemorrhagic presentation (aOR 0.46, 95% CI 0.29–0.73), while statins showed a nonsignificant association (aOR 0.79, 95% CI 0.52–1.20). Randomized propranolol and atorvastatin trials were considered separately because their prospective endpoints and trial populations were not directly comparable with the observational presentation‐based analyses.

**Conclusions:**

Evidence regarding systemic cardiovascular factors and therapies in CCM remains sparse and predominantly of low or very low certainty. The observed associations are preliminary, may reflect residual confounding and treatment‐selection bias, and should not be interpreted as causal or as direct estimates of future hemorrhage risk. Prospective multicenter studies with standardized clinical and imaging definitions are needed before these findings can inform treatment decisions.

## Graphical abstract

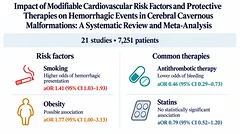

## 1 Introduction

Cerebral cavernous malformations (CCM) are clusters of abnormally dilated capillary channels that may lead to intracerebral hemorrhage, seizures, and focal neurological deficits, resulting in considerable morbidity, particularly among younger adults (Salman et al. 2012, 2016; Santos et al. 2023). Their clinical course is variable, with some lesions remaining asymptomatic while others present with recurrent hemorrhage (Rauscher et al. 2023; Santos et al. 2023; Herten et al. 2021). Although lesion location and prior hemorrhage are established predictors of bleeding risk, identifying which patients are most vulnerable remains a major challenge in clinical practice (Witiw et al. 2012; Santos et al. 2022; Tasiou et al. 2023; Dammann et al. 2025). To that extent, clinicians have assessed possible ways to elucidate that individual risk. For instance, studies have suggested that obesity, smoking, and female hormone therapy intake may increase the likelihood of hemorrhagic events, whereas antithrombotic medications such as aspirin could be associated with decreased hemorrhage (Chen et al. 2023; Flemming et al. 2020; Zuurbier et al. 2023, 2019; Santos et al. 2022; Gomez‐Paz et al. 2020). However, the current evidence is limited to small retrospective series with inconsistent findings and insufficient statistical power (Chen et al. 2023; Flemming et al. 2020; Zuurbier et al. 2023, 2019; Santos et al. 2022; Gomez‐Paz et al. 2020). In the broader cerebrovascular field, both lifestyle and pharmacologic factors are recognized as major determinants of disease risk and outcomes (Hillen et al. 2003; An et al. 2017; Feigin et al. 2005; Karhunen et al. 2021; Macdonald and Schweizer 2017). Although CCM are cerebrovascular lesions, the potential impact of modifiable cardiovascular risk factors, such as smoking, obesity, hypertension, diabetes, and hyperlipidemia, as well as commonly prescribed therapies including antithrombotics and statins has been relatively underexplored. Establishing whether these systemic factors influence hemorrhagic risk could open the door to meaningful strategies for risk reduction and secondary prevention.

To address this knowledge gap, we conducted a systematic review and meta‐analysis assessing associations between modifiable cardiovascular risk factors, systemic therapies, and hemorrhagic outcomes in CCM. We prespecified separate consideration of hemorrhagic presentation at diagnosis and prospective symptomatic hemorrhage or rebleeding, because these endpoints address different clinical questions. Observational estimates were quantitatively synthesized when sufficiently comparable, whereas randomized evidence was interpreted separately when populations or outcomes precluded meaningful pooling.

## 2 Materials and Methods

This systematic review and meta‐analysis were conducted in accordance with the Preferred Reporting Items for Systematic Reviews and Meta‐Analyses (PRISMA) 2020 guidelines and relevant recommendations from the Cochrane Handbook for Systematic Reviews of Interventions. The protocol was prospectively registered in PROSPERO (CRD420251147105).

### 2.1 Literature Search

We conducted a comprehensive search of PubMed/MEDLINE, Embase, and Scopus from database inception through August 31, 2025. Search terms combined “cerebral cavernous malformation,” “cavernoma,” “cavernous angioma,” and “cavernous hemangioma” with hemorrhagic outcomes and prespecified exposures, including smoking, alcohol use, hypertension, diabetes, obesity, lipids, statins, antithrombotic therapy, beta‐blockers, hormone therapy, and pregnancy. The full strategy is presented in Table S1. Reference lists of included articles and relevant reviews were also screened. Human studies in English or with an available English translation were eligible. We included observational cohort and case‐control studies and randomized controlled trials involving adults (≥ 16 years) with CCM and at least one eligible modifiable cardiovascular factor or systemic therapy. Eligible outcomes were hemorrhagic presentation at diagnosis and prospective symptomatic hemorrhage or rebleeding during follow‐up; these outcomes were extracted and interpreted separately. Case reports, reviews, conference abstracts, surgical outcome‐only series, studies limited to radiographic predictors without relevant exposure data, cohorts with fewer than 10 participants, and studies without analyzable or narratively relevant hemorrhagic outcomes were excluded.

### 2.2 Study Selection and Data Collection

Three investigators (S.S., B.D., and J.S.) independently screened titles and abstracts in Rayyan after duplicate removal and then reviewed potentially eligible full texts according to prespecified criteria. Disagreements were resolved by discussion, with a fourth reviewer (A.N.S.) consulted when necessary. Reference lists of included studies and relevant reviews were also screened. The selection process is summarized in the PRISMA flow diagram (Figure 1), and full‐text exclusions are detailed in Table S3.

**FIGURE 1:**
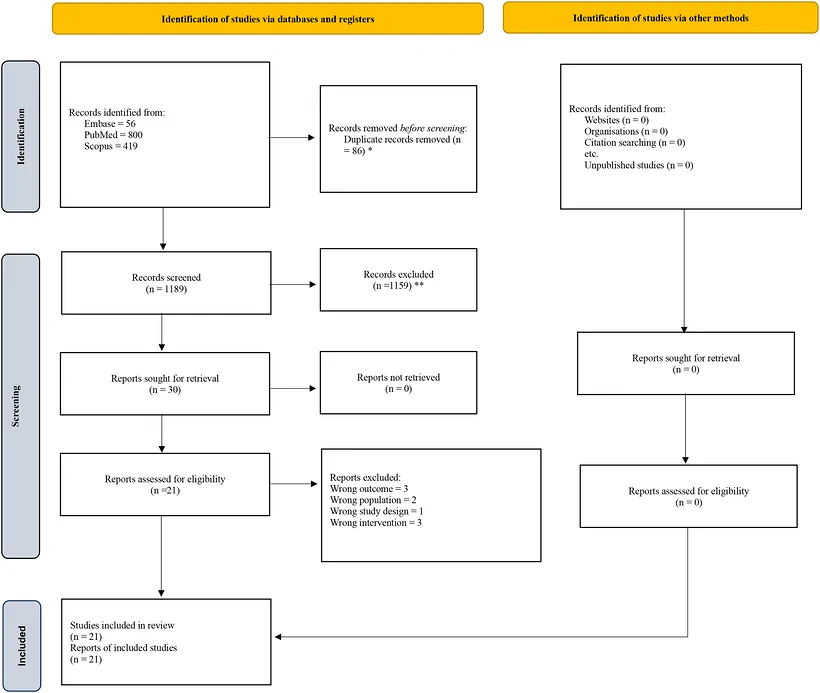
PRISMA flowchart of study selection.

### 2.3 Data Extraction

Two reviewers (S.S. and A.N.S.) independently extracted study design, setting, sample size, demographics, CCM population characteristics when available, cardiovascular risk factors, systemic therapies, hemorrhagic endpoint definition, timing of exposure and outcome, effect estimates, and covariates used in adjusted models. Hemorrhagic presentation at diagnosis was distinguished from prospective symptomatic hemorrhage or rebleeding during follow‐up. We also recorded lesion location, brainstem involvement, prior hemorrhage, multiplicity, familial or sporadic status, genotype, associated developmental venous anomaly, and MRI or hemorrhage definitions when reported. These lesion‐level variables were inconsistently available across studies and therefore could not be incorporated into pooled subgroup analyses. Crude and adjusted estimates were extracted and synthesized separately; no adjusted estimate was combined with an unadjusted estimate in the same model.

### 2.4 Risk of Bias and Certainty Assessment

Risk of bias was assessed independently by two reviewers (S.S. and A.N.S.) using design‐appropriate Cochrane tools. Non‐randomized studies were evaluated with the Risk of Bias in Non‐randomized Studies—of Interventions (ROBINS‐I) tool, while randomized trials were considered using the revised Cochrane risk‐of‐bias tool for randomized trials (RoB 2). GRADE was used to assess the certainty of the body of evidence for each pooled outcome and was not used to conduct the quantitative synthesis. Disagreements were resolved by consensus. Detailed certainty ratings are provided in Table S4.

### 2.5 Statistical Analysis

Associations between eligible exposures and CCM hemorrhagic outcomes were evaluated using crude and adjusted effect estimates in separate analyses. When studies reported adjusted odds ratios (aORs) with 95% confidence intervals (CIs), these were extracted directly. When only event counts were available, 2 × 2 tables were reconstructed and crude odds ratios (ORs) were calculated. Random‐effects models using restricted maximum likelihood were applied to sufficiently comparable datasets. Analyses of hemorrhagic presentation at diagnosis were not interpreted as estimates of future rebleeding. Prospective treatment trials with distinct populations, assigned interventions, or composite outcomes were summarized separately when statistical pooling with observational studies was not clinically appropriate. Heterogeneity was assessed using Cochran's *Q* and *I* ^2^. Meta‐analysis was performed only when at least two independent and sufficiently comparable datasets were available. Publication bias testing was not performed because no pooled analysis contained the approximately 10 or more studies generally required for meaningful funnel‐plot asymmetry assessment. Leave‐one‐out analyses were not performed for comparisons based on only two or three studies because removal of a single study would leave an uninformative estimate. Analyses and plots were generated in R version 4.2.0 using the meta and metafor packages.

## 3 Results

### 3.1 Study Characteristics

Our search yielded 1275 records and 1189 after duplicate removal. Thirty reports were retrieved and assessed for eligibility; eight were excluded, leaving 22 studies in the revised systematic review (Figure 1). These studies included 7331 participants with CCM and comprised retrospective and prospective observational cohorts, case‐control studies, and two randomized controlled trials. The observational studies formed the basis of the pooled analyses when their populations, exposures, and hemorrhagic endpoints were sufficiently comparable. The propranolol Treat_CCM and atorvastatin AT CASH EPOC trials were retained in the qualitative synthesis but interpreted separately because they evaluated assigned therapies in selected populations and used prospective or composite endpoints that were not directly comparable with presentation‐based observational estimates. Table 1 summarizes the included studies and their design classification.

**TABLE 1:** Baseline characteristics of the included studies. Study design is reported as randomized trial, cohort, or case‐control, together with prospective or retrospective data collection when available. Lesion‐level characteristics and adjustment variables were extracted when reported but were too inconsistently available for uniform tabulation.

Study	Study design (period)	Country	Total number of patients	Age, y	Female (%)	Modifiable factors studied	Modifiable factor				
								Exposed		Control	
								Hemorrhage	No hemorrhage	Hemorrhage	No hemorrhage
Searrati et al. (2023)	R, multicenter (NA)	Italy	257	43.4	53.30%	Smoking, diabetes, hypertension, alcohol abuse, antithrombotic therapy, beta‐blockers	Smoking	7 (40)	37 (40)	57 (217)	160 (217)
							Alcohol	2 (5)	3 (5)	63 (252)	189 (252)
							Diabetes	3 (10)	7 (10)	62 (247)	185 (247)
							HTN	6 (66)	60 (66)	59 (191)	132 (191)
							ATT	3 (43)	40 (43)	62 (214)	152 (62)
							BB	3 (36)	33 (36)	62 (221)	159 (221)
Marques et al. (2023)	R, single center (1980–2021)	Switzerland	688	51.3 (±18.7)	46.70%	Antithrombotic therapy, statins	ATT	6 (46)	40 (46)	206 (642)	436 (642)
							Statins	11 (45)	34 (45)	201 (643)	442 (643)
Rauscher et al. (2023)	R, single center (2003–2021)	Germany	89	31.8 (±14.08)	67.40%	Smoking, diabetes, hypertension, obesity, hyperlipidemia	NA	NA	NA	NA	NA
Xiu et al. (2023)	R, single center (2018–2021)	China	120	NA	51.60%	Hyperlipidemia	Hyper‐lipidemia	23 (36)	13 (36)	33 (84)	51 (84)
Chen et al. (2023)	R, single center (2003–2021)	China	1116	42 (±15.9)	61.20%	Antithrombotic therapy, statins, beta‐blockers	NA	NA	NA	NA	NA
Wildi et al. (2023)	R, single center (2006–2018)	Switzerland	428	44.5 (±18.3)	47%	Antithrombotic therapy, statins	ATT	31 (267)	236 (267)	38 (161)	123 (161)
							Statins	20 (267)	247 (267)	26 (161)	135 (161)
Lanfranconi et al. (2023)	RCT, multicenter	Italy	83	45.8 (±14.8)	57.80%	Beta‐blockers	BB	1 (57)	56 (57)	1 (26)	25 (26)
Zuurbier et al. (2022)	P, multi center (1999–2003 or 2006–2010)	Scotland	306	NA	52%	Beta‐blockers, statins	BB	4 (63)	59 (63)	48 (237)	189 (237)
							Statins	13 (73)	60 (73)	39 (227)	118 (227)
Chen et al. (2021)	R, single center (2003–2018)	Germany	682	43 (±13.8)	62.60%	Smoking, diabetes, hypertension, obesity, hyperlipidemia	NA	NA	NA	NA	NA
Gomez‐Paz et al. (2020)	R, single center (1990–2018)	USA	446	50 (± 17)	51.60%	Antithrombotic therapy, statins	ATT	4 (20)	16 (20)	123 (426)	303 (426)
							Statins	12 (54)	42 (54)	115 (392)	227 (392)
Flemming et al. (2020)	P, single center (2015–2018)	USA	202	43.7 (±16.5)	58.40%	Smoking, alcohol, antithrombotic therapy, statins	Smoking	20 (48)	28 (48)	55 (154)	99 (154)
							Alcohol	7 (18)	11 (18)	68 (184)	116 (184)
							ATT	6 (42)	36 (42)	69 (160)	91 (160)
							Statins	9 (34)	25 (34)	66 (168)	102 (168)
Jeon et al. (2014)	R, single center (1998–2010)	South Korea	410	NA	58.50%	Hypertension	HTN	8 (52)	46 (52)	45 (410)	365 (410)
Flemming et al. (2013)	R, single center (1989–1998)	USA	292	NA	42.70%	Antithrombotic therapy	ATT	5 (40)	35 (40)	69 (252)	183 (252)
Schneble et al. (2012)	P, single center (2008–2012)	France	87	44.8 (±17.6)	57.50%	Antithrombotic therapy	ATT	0 (16)	16 (16)	11 (71)	60 (71)
Santos et al. (2022)	R, single center (2003–2020)	Germany	205	34.62 (±18.58)	57.10%	Antithrombotic therapy, statins, beta‐blockers	NA	NA	NA	NA	NA
Bervini et al. (2019)	P, single center (1980–2015)	Switzerland	408	48 (±18.9)	47%	Antithrombotic therapy	ATT	14 (71)	57 (71)	102 (294)	192 (294)
Previch et al. (2022)	P, single center (2015–2021)	USA	364	43.8 (±16)	58%	Antithrombotic therapy, statins, beta‐blockers	ATT	10 (80)	70 (80)	93 (284)	191 (284)
							Statins	20 (91)	71 (91)	83 (273)	190 (273)
							BB	7 (46)	39 (46)	96 (318)	222 (318)
Kong et al. (2022)	P, single center (2009–2013)	China	217	38.3	46.40%	Hypertension	HTN	15 (61)	46 (61)	92 (407)	315 (407)
Guranda et al. (2025)	R, single center (2000–2023)	Germany	143	46.57 (±18.39)	56.60%	Smoking, diabetes, hypertension, antithrombotic therapy,	Smoking	19 (34)	15 (34)	46 (109)	63 (109)
							Diabetes	9 (5)	4 (9)	60 (134)	74 (134)
							HTN	25 (59)	34 (59)	40 (84)	44 (84)
							ATT	5 (24)	19 (24)	60 (119)	59 (119)
Goldberg et al. (2019)	R, single center (1980–2015)	Switzerland	408	50.6 (±18.9)	47.10%	Beta‐blockers	BB	22 (67)	45 (67)	114 (341)	227 (341)
Zuurbier et al. (2019)	P, multi center (1999–2003 or 2006–2010)	Scotland	300	44 (32, 58)	52%	Antithrombotic therapy	ATT	5 (62)	57 (62)	47 (238)	191 (238)
Awad et al. (2025)	RCT, single‐center (2018–2022)	USA	80	41 (median)	63.8%	Statin therapy	Atorvastatin	6 (41)	35 (41)	7 (39)	32 (39)

Abbreviations: ACE, angiotensin‐converting enzyme inhibitors; ATT, antithrombotic therapy; BB, beta‐blockers; CCB, calcium channel blockers; DM, diabetes mellitus; HLD, hyperlipidemia; HTN, hypertension; NA, not available; P, prospective; R, retrospective; RCT, randomized controlled trial; SSRI, selective serotonin reuptake inhibitors.

### 3.2 Risk of Bias and Certainty of Evidence

The pooled certainty assessments primarily reflected non‐randomized evidence and were generally low or very low. ROBINS‐I identified recurring concerns related to confounding and participant selection among the observational studies (Figure S1). The randomized trials were interpreted separately using design‐appropriate considerations because they were not incorporated into the principal pooled estimates. Certainty for each pooled outcome is summarized in Table 2 and Table S4.

**TABLE 2:** Pooled unadjusted and adjusted meta‐analyses of modifiable cardiovascular risk factors and therapies in relation to hemorrhagic presentation in cerebral cavernous malformations. Crude and adjusted estimates were pooled separately. These presentation‐based associations should not be interpreted as estimates of prospective rebleeding.

Modifiable factor	Unadjusted meta‐analysis			Adjusted meta‐analysis			Grade
	No of studies	Pooled OR (95% CI)	*I* ^2^ (%)	No of studies	Pooled OR (95% CI)	*I^2^* (%)	
Smoking or nicotine use	3	1.14 (0.65–2.00)	40.90	3	1.41 (1.03–1.93)	0	⨁⨁◯◯
Diabetes	2	1.41 (0.53–3.71)	0	2	0.83 (0.32–2.13)	0	⨁◯◯◯
Hypertension	4	0.76 (0.35–1.63)	72.80	5	1.02 (0.68–1.53)	0	⨁◯◯◯
Alcohol	2	1.25 (0.52–2.99)	0				⨁◯◯◯
Obesity				2	1.77 (1.00–3.13)	0	⨁◯◯◯
Hyperlipidemia				2	0.51 (0.24–1.09)	0	⨁◯◯◯
Antithrombotic therapy	13	0.35 (0.27–0.45)	0.6	6	0.46 (0.29–0.73)	13.6	⨁⨁◯◯
Beta‐blockers	5	0.45 (0.23–0.88)	50.9	5	0.85 (0.59–1.23)	35.80	⨁◯◯◯
Statins	6	0.65 (0.49–0.85)	0	5	0.79 (0.52–1.20)	0	⨁◯◯◯

*Note*: Grade: ⊕◯◯◯ very low; ⊕⊕◯◯ low; ⊕⊕⊕◯ moderate; ⊕⊕⊕⊕ high.

Abbreviations: aOR, adjusted odds ratio; ATT, antithrombotic therapy; CI, confidence interval; HTN, hypertension; OR, odds ratio.

### 3.3 Smoking

Three studies (Smoking: 122; Non‐smoking: 480 patients) reported unadjusted relation between smoking and the risk of hemorrhage in CCM patients. In the pooled unadjusted analysis 37.7% of patients with a smoking history showed hemorrhage compared to 32.9% in patients with non‐smoking history (OR = 1.14 [95% CI 0.65–2.00] *I* ^2^ = 40.9%). Subgroup analysis based on study design showed no significant association in both prospective (one study) (OR = 1.29 [95% CI 0.66–2.49]) and retrospective (OR = 1.04 [95%CI 0.36–2.95] *I* ^2^ = 69.1%) studies. However, the subgroup differences were not statistically significant (*p* = 0.7321) (Flemming et al. 2020; Guranda et al. 2025; Scerrati et al. 2023) (Figure 2A). Three studies reported adjusted outcomes. In adjusted meta‐analysis, smoking was significantly associated with hemorrhagic presentation in CCM patients (aOR = 1.41 [95%CI 1.03–1.93] *I* ^2^ = 0%) (Rauscher et al. 2023; Gomez‐Paz et al. 2020; Chen et al. 2021; Jeon et al. 2014; Scerrati et al. 2023) (Figure 2B).

**FIGURE 2:**
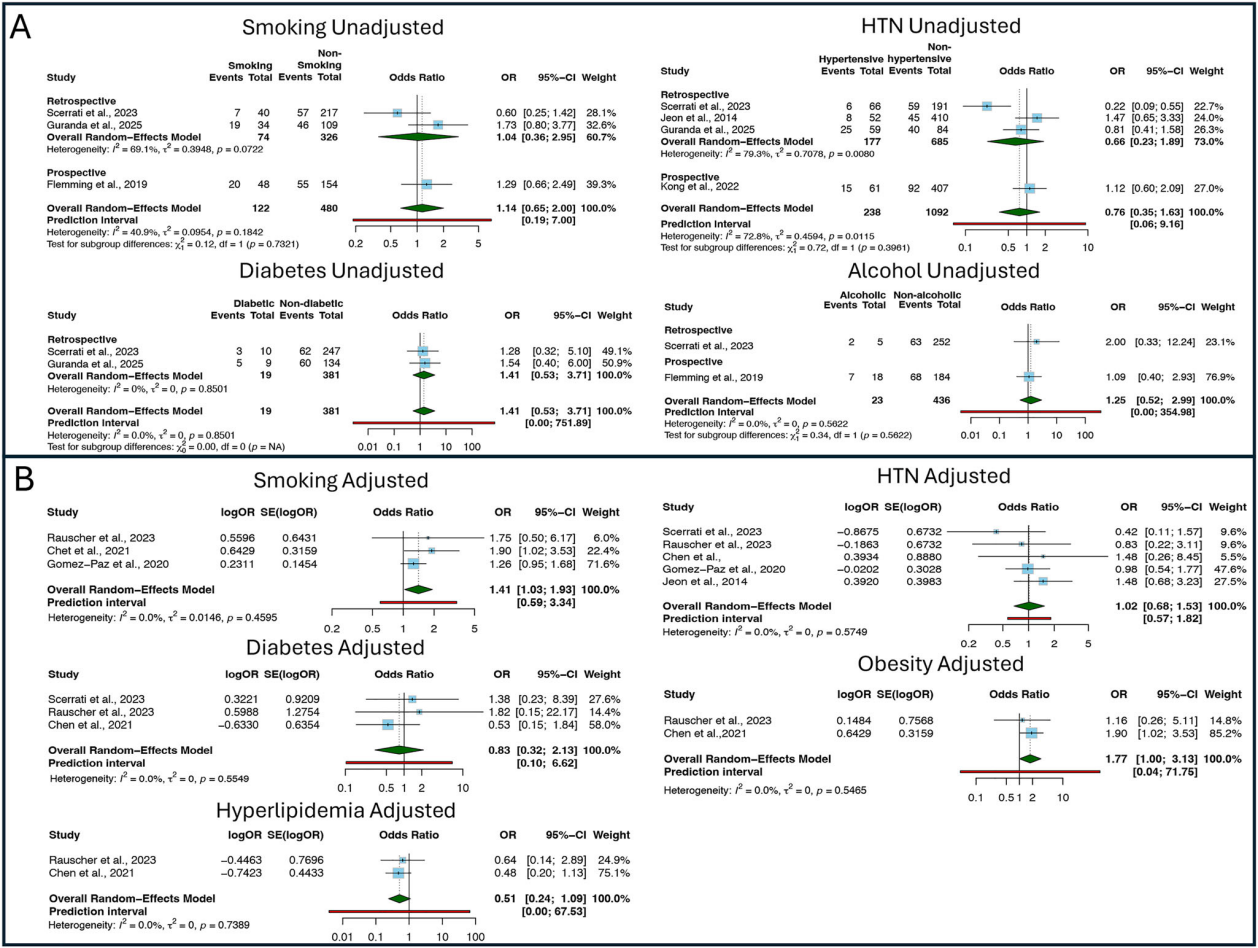
Forest plots of cardiovascular risk factors and first‐presentation hemorrhage in cerebral cavernous malformations. Panel A shows unadjusted associations (smoking, hypertension, diabetes, alcohol); Panel B shows adjusted associations (smoking, hypertension, diabetes, hyperlipidemia, obesity). Effects are random‐effects pooled odds ratios (OR) with 95% confidence intervals; prediction intervals shown where supported. CI, confidence interval; DM, diabetes mellitus; HTN, hypertension; HLD, hyperlipidemia; OR, odds ratio. Adjusted data for alcohol is not available.

### 3.4 Hypertension

Four studies evaluated hypertension (hypertensive: 238; non‐hypertensive: 1092 patients). In pooled unadjusted analysis, 22.6% of hypertensive patients showed hemorrhage compared to 21.6% of non‐hypertensive patients (OR = 0.76 [95% CI 0.35–1.63]; *I* ^2^ = 72.8%). Subgroup analysis showed no significant association in prospective (one study: OR = 1.12 [95% CI 0.60–2.09]) or retrospective designs (OR = 0.66 [95% CI 0.23–1.89]; *I* ^2^ = 79.3%), with no subgroup difference (*p* = 0.3961; Figure 2A) (Jeon et al. 2014; Kong et al. 2022; Guranda et al. 2025; Scerrati et al. 2023). Five retrospective studies reported adjusted outcomes; pooled analysis again showed no significant association (aOR = 1.02 [95% CI, 0.68–1.53]; *I* ^2^ = 0%; Figure 2B) (Rauscher et al. 2023; Gomez‐Paz et al. 2020; Chen et al. 2021; Jeon et al. 2014; Scerrati et al. 2023).

### 3.5 Diabetes

Two retrospective studies (Diabetic: 19; Non‐diabetic: 381) reported unadjusted outcomes. Hemorrhage occurred in 42.1% of diabetics versus 32.0% of non‐diabetics (OR = 1.14 [95% CI 0.53–3.71]; *I* ^2^ = 0%; Figure 2A) (Guranda et al. 2025; Scerrati et al. 2023). Three studies reported adjusted outcomes, with pooled analysis showing no significant association (aOR = 0.83 [95% CI 0.32–2.13]; *I* ^2^ = 0%; Figure 2B) (Rauscher et al. 2023; Gomez‐Paz et al. 2020; Chen et al. 2021).

### 3.6 Obesity

Two studies reported adjusted outcomes. Pooled analysis showed obesity was significantly associated with hemorrhagic presentation (aOR = 1.77 [95% CI 1.00–3.13]; *I* ^2^ = 0%; Figure 2B) (Rauscher et al. 2023; Chen et al. 2021). The CI barely excluded 1, suggesting a possible but uncertain association.

### 3.7 Hyperlipidemia

Two studies reported adjusted outcomes. Pooled analysis showed no significant association between hyperlipidemia and hemorrhagic presentation (aOR = 0.51 [95% CI 0.24–1.09]; *I* ^2^ = 0%; Figure 2B).

### 3.8 Alcohol

Two studies (Alcoholic: 23; Non‐alcoholic: 436) reported unadjusted outcomes. Hemorrhage occurred in 39.1% of alcohol users versus 30.0% of non‐users (OR = 1.25 [95% CI 0.52–2.99]; *I* ^2^ = 0%; Figure 2A) (Flemming et al. 2020; Scerrati et al. 2023).

### 3.9 Beta‐Blockers

Five studies reported beta‐blocker exposure. The observational estimates and the Treat_CCM randomized trial differed in design, population, and outcome definition. Treat_CCM enrolled patients with symptomatic familial CCM and evaluated a composite of symptomatic intracerebral hemorrhage or a new focal neurological deficit; it was therefore interpreted separately rather than as direct evidence regarding hemorrhagic presentation. Across the observational adjusted analyses, beta‐blocker use was not significantly associated with hemorrhagic presentation (aOR = 0.85 [95% CI 0.59–1.23]; *I* ^2^ = 35.8%; Figure 3B).

**FIGURE 3:**
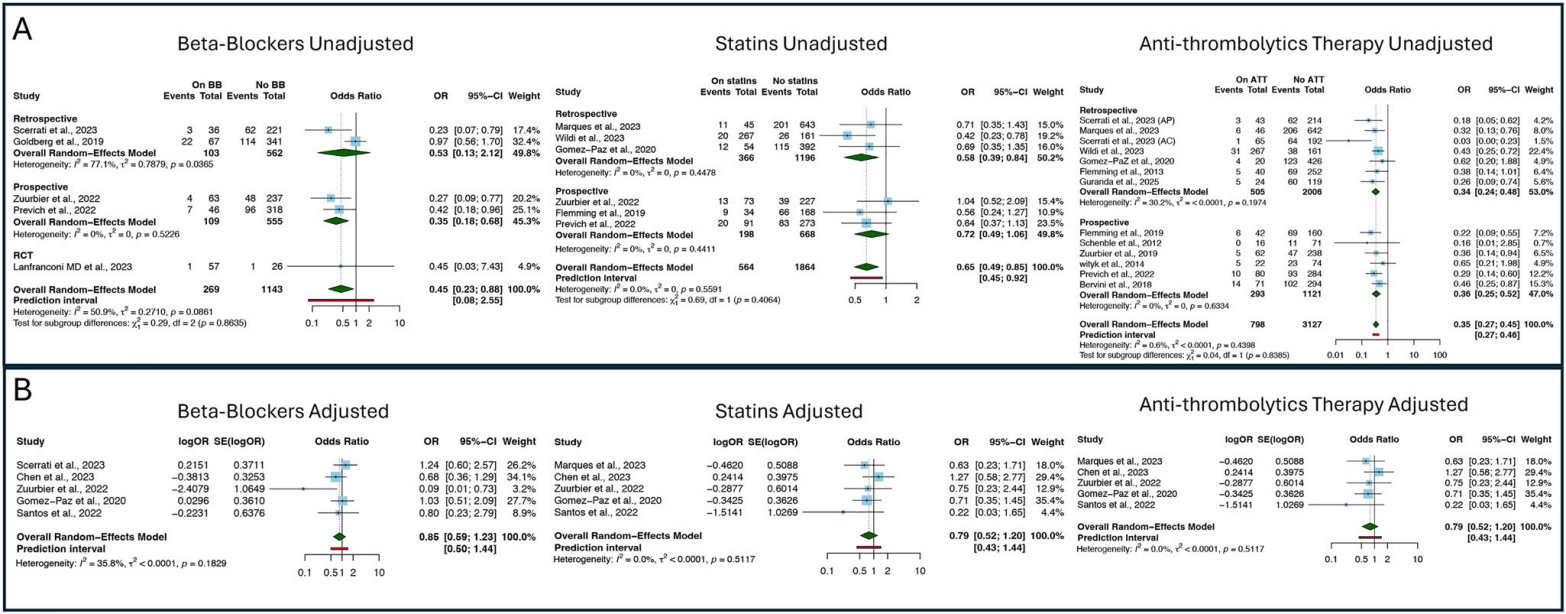
Forest plots of pharmacological therapies and first‐presentation hemorrhage. Panel A shows unadjusted associations; Panel B shows adjusted associations for the same therapies. Effects are random‐effects pooled odds ratios (OR) with 95% confidence intervals; prediction intervals shown where supported. ATT, antithrombotic therapy; BB, beta‐blockers; CI, confidence interval; OR, odds ratio.

### 3.10 Statins

Six observational studies reported statin exposure in relation to hemorrhagic presentation. The pooled unadjusted estimate suggested lower odds among statin users (OR = 0.65 [95% CI 0.49–0.85]; *I* ^2^ = 0%), whereas the adjusted analysis was not significant (aOR = 0.79 [95% CI 0.52–1.20]; *I* ^2^ = 0%; Figure 3). The AT CASH EPOC randomized trial was added to the qualitative synthesis but not pooled because it enrolled patients after a recent symptomatic hemorrhage and evaluated prospective rebleeding, with an imaging biomarker as its primary efficacy endpoint. Symptomatic hemorrhage occurred in 6 of 41 atorvastatin participants and 7 of 39 placebo participants (RR 0.81, 95% CI 0.31–2.13), providing no conclusive evidence of benefit or harm.

### 3.11 Antithrombotic Therapy

Observational studies reported antithrombotic exposure at different time points, including medication use at diagnosis and use during follow‐up. In pooled presentation‐based analyses, antithrombotic therapy was associated with lower odds of hemorrhagic presentation in both crude and adjusted models. This association should not be interpreted as proof of a protective drug effect because treatment may have been withheld from patients perceived to have higher‐risk lesions or recent hemorrhage. Antiplatelet and anticoagulant agents could not be consistently separated across the source studies. The adjusted estimate was aOR = 0.46 (95% CI 0.29–0.73; *I* ^2^ = 13.6%; Figure 3B).

## 4 Discussion

CCM hemorrhage is primarily influenced by lesion‐level and clinical characteristics, including brainstem or deep location, prior symptomatic hemorrhage, multiplicity, familial or genetic status, associated developmental venous anomaly, and imaging phenotype. Against that background, this review examined whether systemic cardiovascular factors and commonly prescribed therapies were associated with hemorrhagic outcomes. The available evidence was predominantly retrospective and heterogeneous in endpoint definition and adjustment strategy. Most pooled analyses concerned hemorrhagic presentation at diagnosis rather than prospective rebleeding; therefore, the estimates should not be interpreted as direct predictions of future hemorrhage. Smoking and obesity showed possible associations with hemorrhagic presentation, whereas antithrombotic exposure was associated with lower odds in observational analyses. These findings remain preliminary and potentially confounded.

Smoking was associated with higher odds of hemorrhagic presentation in the adjusted analysis, but this finding was based on only a few observational datasets and low‐certainty evidence. Low statistical heterogeneity does not eliminate the possibility of residual confounding, differences in lesion phenotype, or selection bias. The result should therefore be regarded as a hypothesis‐generating association rather than a robust or lesion‐independent predictor.

Obesity showed an uncertain association based on only two studies. Differences in genetic background, lesion burden, location, prior hemorrhage, and referral patterns may account for variation between cohorts, particularly when familial CCM1 populations are compared with sporadic or unselected CCM cohorts. The available data do not support incorporating obesity into clinical risk models at present.

In contrast, hypertension, diabetes, and hyperlipidemia, despite being well‐recognized drivers of stroke and other cerebrovascular conditions (O'Donnell et al. 2010; Vanent et al. 2025; Korja et al. 2013), did not demonstrate associations with CCM hemorrhage. These null results may reflect differences in the underlying pathophysiology of CCM compared with other vascular diseases (Snellings et al. 2021). Alternatively, the lack of effect could be explained by insufficient statistical power, variation in exposure definitions, or incomplete adjustment for confounding variables across included studies.

The observational association between antithrombotic therapy and lower odds of hemorrhagic presentation requires particular caution. Patients with recent hemorrhage, brainstem lesions, repeated bleeds, or other high‐risk imaging features may be less likely to receive or continue antithrombotic treatment, creating selection bias and reverse causation. Exposure timing and separation of antiplatelet from anticoagulant therapy were also inconsistent. Proposed mechanisms involving intralesional thrombosis or venous congestion remain speculative, especially because associated developmental venous anomaly status was rarely reported. Similarly, the adjusted observational statin estimate was nonsignificant, and the AT CASH EPOC trial did not establish a reduction in prospective symptomatic hemorrhage. Neither therapy should be described as protective on the basis of the current evidence.

### 4.1 Limitations

This analysis has several limitations. Most pooled comparisons were supported by few observational datasets and were at a moderate or serious risk of bias. Hemorrhagic presentation at diagnosis and prospective symptomatic hemorrhage or rebleeding were not uniformly defined across the literature and address different clinical questions. Diagnostic MRI sequences and distinctions between symptomatic acute hemorrhage, chronic hemosiderin deposition, and silent prior bleeding were inconsistently reported. Key CCM‐specific determinants—including brainstem or deep location, prior hemorrhage, multiplicity, familial or sporadic disease, genotype, lesion burden, associated developmental venous anomaly, lesion size, and MRI phenotype—were incompletely reported and inconsistently included in adjusted models. Medication timing, dose, duration, and prescribing indications were also variable, creating potential confounding by indication and treatment‐selection bias. These limitations precluded lesion‐level subgroup analyses and restrict external validity. Accordingly, the findings should be interpreted as preliminary and hypothesis‐generating.

The revised synthesis therefore emphasizes uncertainty rather than clinical actionability. Standardized prospective cohorts and adequately powered randomized trials using consensus definitions of symptomatic hemorrhage, detailed lesion‐level imaging characterization, and prespecified adjustment strategies are needed before systemic factors or therapies can be incorporated into CCM prognostic models or preventive recommendations.

## 5 Conclusion

Current evidence suggests possible associations between selected systemic factors or therapies and hemorrhagic presentation in CCM, but it does not establish causality or reliably estimate prospective rebleeding risk. Smoking and obesity showed uncertain associations with higher odds of hemorrhagic presentation, while observational antithrombotic findings may be strongly influenced by treatment‐selection bias. Adjusted statin and beta‐blocker estimates were inconclusive, and the available randomized trials were not definitive. Given the low or very low certainty of evidence and incomplete adjustment for lesion‐level risk determinants, these results should remain hypothesis‐generating until validated in prospective multicenter studies.

## Author Contributions

Concept and design: **Sai Sanikommu**, **Alejandro N. Santos**, and **Robert M. Starke**. Literature search: Sai Sanikommu, Alejandro N. Santos, **Bashar Dawoud**, **Victor M. Lu**, **Jayro Toledo**, **Emade Jaman**, Josiah J. Z. Sherman, **Alick Wang** and **Ahmed Abdelsalam**. Statistical analysis: Sai Sanikommu. Figures and Tables: Sai Sanikommu, Alejandro N. Santos, Bashar Dawoud, Victor M. Lu, Jayro Toledo, Emade Jaman, Josiah J. Z. Sherman, Alick Wang and Ahmed Abdelsalam. Drafting the first manuscript: Sai Sanikommu and Alejandro N. Santos. Revision of the manuscript for important intellectual content: Sai Sanikommu, Robert M. Starke, and **Christoph Wipplinger**. Approval of final manuscript version for submission: All authors.

## Funding

Robert M. Starke research is supported by the Codina Kadre Endowed Chair in Neurosurgery, the NREF, Joe Niekro Foundation, Brain Aneurysm Foundation, Bee Foundation, TAAF, Florida Department of Health James and Esther King Biomedical Research Program (21K02), and by the National Institutes of Health (Grant numbers: R01NS111119‐01A1, UL1TR002736, KL2TR002737) through the Miami Clinical and Translational Science Institute. Its contents are solely the responsibility of the authors and do not necessarily represent the official views of the NIH.

## Ethics Statement

Ethical approval was not required for this study because it is a systematic review and meta‐analysis of previously published studies and did not involve the collection of new data from human participants or animals.

## Conflicts of Interest

Robert M. Starke reports consulting and teaching agreements with Penumbra, Abbott, Balt, Kaneka, Medtronic, Stryker, Microvention, Integer PPF, InNeuro Co, VonVascular, Optimize Vascular, and Cerenovus. The other authors declare no conflicts of interest.

## Supporting information




**Supporting Information**: brb371829‐sup‐0001‐SuppMat.docx

## Data Availability

The data that support the findings of this study are available upon reasonable request from the corresponding author.
