# Supplementary material for: Impact of Modifiable Cardiovascular Risk Factors and Protective Therapies on Hemorrhagic Events in Cerebral Cavernous Malformations: A Systematic Review and Meta‐Analysis
Source: Brain Behav. 2026 Oct 8;16(10):e71829. doi: 10.1002/brb3.71829 (PMC13650498; doi:10.1002/brb3.71829)
Supplement: Supplementary file 1 [file BRB3-16-e71829-s001.docx]

**Supplementary Table 1. Search strategy**

| Database |  | Results | | |
| --- | --- | --- | --- | --- |
| PUBMED/MEDLINE | #1 ("cerebral cavernous malformation" OR cavernoma OR "cavernous angioma" OR "cavernous hemangioma" OR CCM) | 800 | | |
|  | **#2** AND |  |  |  |
|  | **#3** (hemorrhage OR bleeding OR heamorrhage OR "focal neurological deficit") |  |  |  |
|  | **#4** #1AND#2 AND #3 |  |  |  |
|  | **#5** (smoking OR alcohol OR hypertension OR diabetes OR obesity OR BMI OR cholesterol OR lipids OR contraceptive OR estrogen OR "hormone therapy" OR antithrombotic OR aspirin OR statin OR pregnancy) |  |  |  |
| EMBASE | #1 ('cerebral cavernous malformation'/exp OR cavernoma:ti,ab OR "cavernous hemangioma":ti,ab OR CCM:ti,ab) | 56 |  |  |
|  | #**2** AND |  |  |  |
|  | **#3** Hemorrhage/exp OR bleeding:ti,ab OR "focal neurological deficit":ti,ab) |  |  |  |
|  | **#4** #1AND #2 AND #3 |  |  |  |
|  | **#5** (smoking/exp OR alcohol/exp OR hypertension/exp OR diabetes mellitus/exp OR obesity/exp OR cholesterol/exp OR contraceptive agent/exp OR estrogen/exp OR hormone therapy/exp OR antithrombotic agent/exp OR aspirin:ti,ab OR statin:ti,ab OR pregnancy/exp) |  |  |  |
|  | **#6** NOT ([animals]/lim NOT [humans]/lim) |  |  |  |
| Scopus | #1 TITLE-ABS-KEY ("cerebral cavernous malformation" OR cavernoma OR "cavernous angioma" OR "cavernous hemangioma" OR CCM) | 419 |  |  |
|  | **#2** AND |  |  |  |
|  | **#3** TITLE-ABS-KEY (hemorrhage OR bleeding OR heamorrhage OR "focal neurological deficit") |  |  |  |
|  | **#4** #1AND#2 AND #3 |  |  |  |
|  | **#5** TITLE-ABS-KEY (smoking OR alcohol OR hypertension OR diabetes OR obesity OR BMI OR cholesterol OR lipids OR contraceptive OR estrogen OR "hormone therapy" OR antithrombotic OR aspirin OR statin OR pregnancy) |  |  |  |

**Supplementary Table 2. Inclusion and exclusion criteria for study eligibility.**

|  | Inclusion Criteria | Exclusion Criteria |
| --- | --- | --- |
| Population | - Adult patients (≥16 years) with confirmed diagnosis of CCM and with history of ICH due to CMM recorded. Eligible study designs included observational cohort or case-control studies and randomized controlled trials with relevant hemorrhagic outcomes. Hemorrhagic presentation and prospective symptomatic hemorrhage/rebleeding were extracted separately. | - Children and adolescent patients (< 16 years old). - Patients with no recorded history of ICH due to CCM. - Patients with spinal CM |
| Intervention | - Patients who had CCM and history of hemorrhage with at-least one modifiable factor recorded (Smoking or nicotine use, Diabetes, Hypertension, Hyperlipidemia, Obesity, Antithrombotic therapy, Beta-blockers, Statins) |  |
| Comparator | - Patients who had CCM and history of hemorrhage without any above-mentioned modifiable factors |  |

CCM: Cerebral cavernous malformations; ICH: Intracerebral hemorrhage

**Supplementary Table 3. Table of excluded studies.**

| Author | Title | Exclusion Reason |
| --- | --- | --- |
| Padarti et al., | Readmissions in patients with cerebral cavernous malformations: a national readmission database study | Wrong study design |
| Zuurbier et al., | Female Hormone Therapy and Risk of Intracranial Hemorrhage from Cerebral Cavernous Malformations: A Multicenter Observational Cohort Study | Wrong population |
| Choquet et al., | Association of cardiovascular risk factors with disease severity in cerebral cavernous malformation type 1 subjects with the common Hispanic mutation | Wrong outcome |
| Salman et al., | Metabolic Syndrome and Hemorrhagic Stroke in Hispanic Elderly Patients with Cerebral Cavernous Malformations | Wrong intervention |
| Vysakhs et al., | Risk of Bleed During Pregnancy in Women with Epilepsy Due to Cerebral Vascular Malformations | Wrong population |
| Pei et al., | Clinical characteristics and management of cerebral developmental venous anomalies accompanied with cavernous malformation | Wrong outcome |
| Zou et al., | Predictors of postoperative epileptic seizures after microsurgical treatment in supratentorial single cerebral cavernous malformations: a retrospective study | Wrong intervention |
| Mattei et al., | Treating head and neck venous malformations with cold helium plasma electrosurgical device: A 17 patients case series | Wrong intervention |

The AT CASH EPOC atorvastatin trial was added to the revised qualitative synthesis. It was not pooled with observational presentation-based analyses because it evaluated assigned treatment after recent symptomatic hemorrhage and reported prospective rebleeding, with QSM change as the primary efficacy endpoint.

**Supplementary Table 4.** Certainty of evidence of the included studies

| Factor | No. of studies | aOR (95% CI) | I² | Risk of Bias | Inconsistency | Imprecision | Certainty |
| --- | --- | --- | --- | --- | --- | --- | --- |
| Smoking | 3 | 1.41 (1.03–1.93) | 0% | Serious^a^ | Not serious | Not serious | ⨁⨁◯◯ Low |
| Diabetes | 2 | 0.83 (0.32–2.13) | 0% | Serious^a^ | Not serious | Serious ^c^ | ⨁◯◯◯ Very Low |
| Hypertension | 5 | 1.02 (0.68–1.53) | 0% | Serious^a^ | Not serious | Serious ^c^ | ⨁◯◯◯ Very Low |
| Alcohol | 2 | – | – | Serious^a^ | – | Serious ^c^ | ⨁◯◯◯ Very Low |
| Obesity | 2 | 1.77 (1.00–3.13) | 0% | Serious^a^ | Not serious | Serious ^b^ | ⨁◯◯◯ Very Low |
| Hyperlipidemia | 2 | 0.51 (0.24–1.09) | 0% | Serious^a^ | Not serious | Serious ^c^ | ⨁◯◯◯ Very Low |
| Antithrombotic therapy | 6 | 0.46 (0.29–0.73) | 13.6% | Serious^a^ | Not serious | Not serious | ⨁⨁◯◯ Low |
| Beta-blockers | 5 | 0.85 (0.59–1.23) | 35.8% | Serious^a^ | Not serious | Serious ^c^ | ⨁◯◯◯ Very Low |
| Statins | 5 | 0.79 (0.52–1.20) | 0% | Serious^a^ | Not serious | Serious ^c^ | ⨁◯◯◯ Very Low |

^a^We downgraded the certainty of evidence by one level due to serious concerns. This decision was based on domains as selection of participants, classification of the interventions, and selection of the reported result. These biases can affect the internal validity of the studies and, consequently, the robustness of the pooled effect estimate.

^b^ We downgraded one level in imprecision. The confidence intervals are wide, indicating uncertainty in the effect estimate. Additionally, the sample size is not sufficient to provide precise results.

^c^We downgraded two levels in imprecision The confidence interval crosses the null value, indicating uncertainty about the direction of the effect. The sample size and number of events are insufficient, leading to a lack of statistical power.

**Supplementary Figure 1**. RRisk of bias was assessed using the ROBINS-I tool across seven domains: bias due to confounding (D1), selection of participants (D2), classification of interventions (D3), deviations from intended interventions (D4), missing data (D5), measurement of outcomes (D6), and selection of the reported result (D7). Each study was graded as low (green), moderate (yellow), or serious risk (red) in each domain, with an overall judgment provided in the final column. Most studies were rated at low to moderate risk overall, with frequent concerns regarding confounding and participant selection. Outcome measurement was consistently rated low risk. Several studies were judged at serious overall risk of bias, reflecting limitations in design and adjustment, underscoring the observational nature of the available evidence.
